# miR-375 inhibits the proliferation of gastric cancer cells by repressing ERBB2 expression

**DOI:** 10.3892/etm.2014.1627

**Published:** 2014-03-20

**Authors:** ZHI-YONG SHEN, ZI-ZHEN ZHANG, HUA LIU, EN-HAO ZHAO, HUI CAO

**Affiliations:** Department of General Surgery, Renji Hospital, School of Medicine, Shanghai Jiaotong University, Shanghai 201112, P.R. China

**Keywords:** gastric cancer, microRNA, human epidermal growth factor receptor 2, proliferation

## Abstract

MicroRNAs (miRNAs) are small non-coding RNA molecules that regulate the expression of targeted genes in a post-transcriptional manner. Increasing evidence indicates that miRNAs play important roles in cancer pathogenesis, including apoptosis, proliferation and differentiation, as oncogenes or tumor suppressors. Previously, miR-375 was shown to be involved in human gastric cancer, however, the mechanism remains poorly understood. In the present study, miR-375 was shown to be downregulated in gastric cancer tissues, particularly human epidermal growth factor receptor 2 (ERBB2)-positive gastric cancer tissues. Identified by dual luciferase assays and western blot analysis, ERBB2 was demonstrated to be a target gene of miR-375. In addition, miR-375 overexpression suppressed the proliferation of human gastric cancer cells *in vitro* and the suppression effect was restored by ERBB2 overexpression. Thus, the results of the present study indicate that miR-375 is associated with human gastric carcinogenesis by targeting ERBB2. Therefore, miR-375 may be used as a potential clinical classification marker and therapeutic target for human gastric cancer.

## Introduction

Gastric cancer is the fourth most common type of cancer causing ~800,000 mortalities worldwide each year (1). In general, gastric cancer has a 5-year survival rate of ~15% and for patients with advanced gastric cancer, the median overall survival is <1 year (2,3). Considering these statistics, increased research into potential preventative methods for gastric cancer is required, as well as improved early detection and more effective treatments.

Human epidermal growth factor receptor 2 (ERBB2) is an important biomarker not only for breast cancer, but for other types of cancer prognosis and patient treatment decisions as well. ERBB2 is a member of the epidermal growth factor receptor (EGFR) family that is associated with increased proliferation of tumor cells. As determined by various studies, between 6 and 35% of patients with gastric and gastroesophageal junction cancers exhibit ERBB2 gene amplification and protein overexpression (3–6).

MicroRNAs (miRNAs) are short non-coding RNA molecules that suppress the expression of protein coding genes by partial complementary binding, particularly to 3′ untranslated regions (UTRs) of mRNAs. Alterations in miRNA expression are involved in the initiation, progression and metastasis of human cancer and it has been hypothesized that miRNAs function as tumor suppressors and oncogenes in cancer development (7,8). Based on 160 paired samples of non-tumor mucosa and cancer cells, Ueda *et al* reported that 22 miRNAs were upregulated and 13 miRNAs were downregulated in gastric cancer, indicating that specific miRNAs are associated with the progression and prognosis of gastric cancer (9).

A number of studies have revealed that the expression of miR-375 is reduced in several human cancers, including head and neck squamous cell carcinoma, esophageal cancer and hepatocellular carcinoma (10–12). In addition, previous studies have indicated that miR-375 may be one of the most important miRNAs involved in the progression of gastric cancer (13,14). Therefore, in the present study, the expression and mechanisms of miR-375 were investigated in gastric cancer with the aim of providing a novel candidate for the diagnosis and treatment of human gastric cancer.

## Materials and methods

### Tissue samples

For miR-375 detection, 30-paired gastric tissue samples were collected (cancer lesions and adjacent non-tumor mucosae) from patients that had undergone gastrectomy at Renji Hospital (Shanghai, China) from March 2011 to January 2013. All the samples were collected in the same manner and snap-frozen immediately in liquid nitrogen. The samples were stored at −80°C until required for RNA and protein extraction. Since microdissection is difficult to perform in diffuse-type gastric cancer, bulk tissue was used in all the cases for technical uniformity. Approval for the study was provided by the Ethics Committee of Renji Hospital and every patient provided written informed consent. Diagnosis of gastric cancer was confirmed by at least two pathologists and staging was based on pathological observations according to the 7th American Joint Committee on Cancer guidelines (15).

### Gastric cancer cell lines

The BGC-823 human gastric adenoma cell line was purchased from the Cell Bank of Shanghai (Shanghai, China). Cells were routinely cultured in RPMI 1640 medium, supplemented with 10% fetal bovine serum (Hyclone, Logan, UT, USA), at 37°C in a humidified atmosphere with 5% CO_2_.

### ERBB2 expression vector construction

The full length coding region of human ERBB2 was amplified by reverse transcription polymerase chain reaction (PCR) and cloned into the pcDNA3.1 vector (Invitrogen, Carlsbad, CA, USA), which was then designated as pcDNA3.1-ERBB2. This vector and the control vector, pcDNA3.1, were transfected into cells using Lipofectamine 2000 (Invitrogen Life Technologies, Carlsbad, CA, USA), according to the manufacturer’s instructions.

### 3′-UTR luciferase reporter assays

To generate the 3′-UTR luciferase reporter, the full length of the 3′-UTR from ERBB2 was cloned into the downstream region of the firefly luciferase gene using the pGL3-control vector (Promega Corporation, Madison, WI, USA). Mutant miR-375 target sites in the 3′-UTR of ERBB2 were used as corresponding controls. An miR-375 mimic and inhibitor were synthesized by Shanghai GenePharma Co., Ltd (Shanghai, China) and a pRL-TK plasmid (Promega), containing *Renilla* luciferase, was cotransfected for data normalization. For the luciferase reporter assays, BGC-823 cells were seeded in 48-well plates. Luciferase reporter vectors were cotransfected with miR-375 mimic or miR-375 inhibitor using Lipofectamine 2000. After two days, the cells were harvested and assayed with the Dual-Luciferase Assay (Promega Corporation). Experiments were performed in triplicate and the results are expressed as relative luciferase activity (Firefly luciferase activity/*Renilla* luciferase activity).

### Western blot analysis

Protein extracts were boiled in SDS/β-mercaptoethanol sample buffer, and 30-μg protein samples were loaded into each lane of the 8% polyacrylamide gels. Proteins were separated by electrophoresis and then blotted onto polyvinylidene fluoride membranes (Amersham Pharmacia Biotech, Amersham, UK) by electrophoretic transfer. The membranes were incubated with mouse anti-ERBB2 (Abcam, Cambridge, MA, USA) or mouse anti-β-actin monoclonal antibodies (Santa Cruz Biotechnology, Inc., Santa Cruz, CA, USA) for 1 h at 37°C. Specific protein-antibody complexes were then detected using horseradish peroxidase-conjugated rabbit anti-mouse secondary IgG. Detection was performed using an enhanced chemiluminescence kit (Pierce Manufacturing, Inc., Appleton, WI, USA) and the β-actin signal was used as a loading control.

### RNA extraction and miR-375 expression detection

Quantitative PCR analysis was used to determine the relative expression levels of miR-375. Total RNA was extracted from the tissue samples using TRIzol reagent (Invitrogen Lift Technologies), according to the manufacturer’s instructions. The expression level of miR-375 was detected using *Taq*Man miRNA quantitative PCR. Single-stranded cDNA was synthesized using a *Taq*Man microRNA reverse transcription kit (Applied Biosystems, Inc., Foster City, CA, USA), which was then amplified using *Taq*Man Universal PCR Master Mix (Applied Biosystems, Inc.) with miRNA-specific *Taq*Man minor groove binder probes (Applied Biosystems, Inc.). U6 spliceosomal RNA was used for normalization. Each sample was measured in triplicate for the detection of miR-375 expression.

### Cell proliferation assays

BGC-823 cells were seeded in 96-well plates at a low density of 5×10^3^ cells/well in Dulbecco’s modified Eagle’s medium (Hyclone) and allowed to attach overnight. The cells were then transfected with miR-375 mimic or miR-375 mimic plus pcDNA3.1-ERBB2, with nonsense short RNA and miR-375 plus pcDNA-3.1 used as controls. Next, 20 μl MTT (5 mg/ml; Sigma-Aldrich, St. Louis, MO, USA) was added to each well 48 h following transfection and the cells were incubated for a further 4 h. Absorbance was recorded at 570 nm with a 96-well plate reader following the addition of dimethyl sulfoxide.

### Statistical analysis

Data were analyzed using SPSS software, version 16 (SPSS, Inc., Chicago, IL, USA). Results from the independent groups were analyzed using the t-test. Tissue miR-375 expression levels were analyzed using the Mann-Whitney U-test. P<0.05 was considered to indicate a statistically significant difference.

## Results

### miR-375 is downregulated in gastric cancer tissues, particularly ERBB2-positive tissues

**Figure 1:**
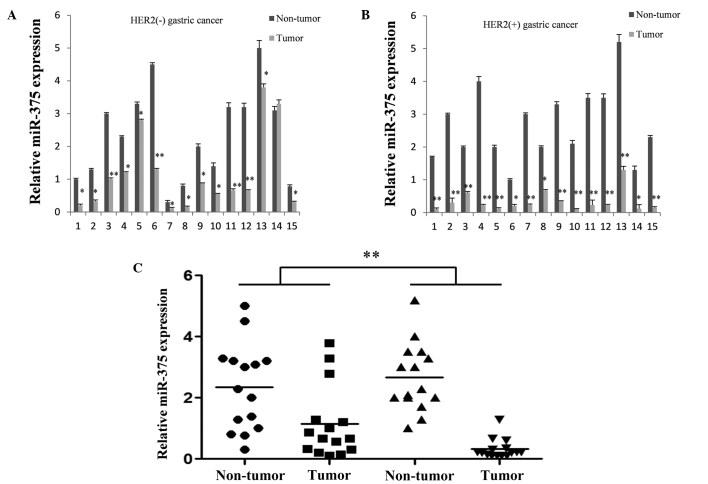
Expression levels of miR-375 were downregulated in gastric cancer, particularly ERBB2-positive gastric cancer tissues. Detection of miR-375 expression levels in normal and (A) ERBB2-negative and (B) -positive gastric cancer tissue samples. (C) Comparison between miR-375 expression levels in ERBB2-negative and -positive gastric cancer tissues. ERBB2, human epidermal growth factor receptor 2; miR-375, microRNA-375. ^*^P<0.05, ^**^P<0.01.

Quantitative PCR was used to compare the expression levels of miR-375 among 30 cases of normal and gastric cancer tissue samples. Each tumor and normal sample was derived from a single patient specimen. For the majority of cases, the expression level of miR-375 was significantly decreased in the gastric cancer tissues when compared with the corresponding non-cancerous tissues (Fig. 1A and B). In addition, miR-375 expression levels were markedly reduced in ERBB2-positive gastric cancer tissues (Fig. 1C).

### ERBB2 is the target gene of miR-375

**Figure 2:**
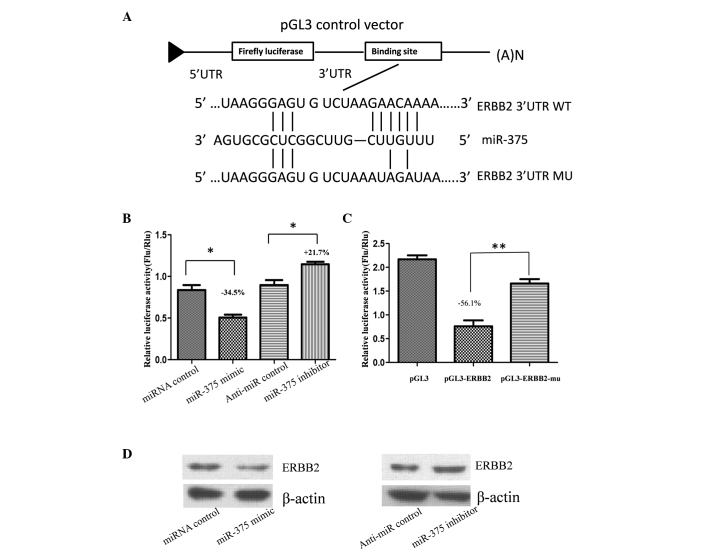
ERBB2 is a target gene of miR-375. (A) Schematic diagram for constructing the predicted miR-375 binding site in the pGL3 control vector. (B) Confirmation that ERBB2 is the target gene of miR-375. BGC-823 cells were cotransfected with pGL3-ERBB2 and miRNA control, miR-375 mimic, anti-miR control or miR-375 inhibitor for the dual-luciferase assays. A pRL-TK plasmid containing *Renilla* luciferase was cotransfected with the 3′-UTR of ERBB2 for data normalization. (C) Mutation analysis of the miR-375 binding site. When four nucleotides in the binding site of miR-375 in the 3′-UTR of ERBB2 were mutated (pGL3-ERBB2-Mu), luciferase activity significantly decreased in the BGC-823 cells that had been cotransfected with miR-375 mimic and pGL3-ERBB2, as compared with cells transfected with pGL3-ERBB2-Mu or pGL3. (D) ERBB2 protein expression levels in miR-375 mimic or inhibitor-treated BGC-823 cells, as detected by western blot analysis. ERBB2, human epidermal growth factor receptor 2; miR-375, microRNA-375; UTR, untranslated region. ^*^P<0.05, ^**^P<0.01.

miRNA is an important post-transcriptional negative regulator for protein coding genes. Thus, to explore the association between reduced miR-375 expression levels and ERBB2 overexpression, miR-375 targets were predicted using the online bioinformatic tools, TargetScan (http://www.targetscan.org/) and miRanda (http://www.microrna.org/microrna/home.do). According to the results of the online prediction, miR-375 targets ERBB2 directly. Therefore, to validate whether ERBB2 is the target gene of miR-375, the full length of the 3′-UTR of human ERBB2 was cloned into the downstream region of the firefly luciferase reporter gene using the pGL3 control vector (pGL3-ERBB2) for the dual luciferase assay (Fig. 2A). Human gastric adenoma BGC-823 cells were cotransfected with pGL3-ERBB2 and miR-375 mimic or inhibitor (Fig. 2B). Compared with the miRNA control, luciferase activity was significantly suppressed with miR-375 by ~34.5% (P<0.05). Furthermore, luciferase activity was significantly upregulated by ~21.7% (P<0.05) with the miR-375 inhibitor, as compared with the anti-miR control. These results indicate that miR-375 targets the 3′-UTR of ERBB2, leading to a change in firefly luciferase translation.

A seed sequence mutation clone was also used to further confirm the binding site for miR-375 (Fig. 2A). A putative miR-375 binding region in the 3′-UTR of ERBB2 with four mutant nucleotides (pGL3-ERBB2-Mu) and the pGL3 empty vector were used as controls. The histogram in Fig. 2C indicates that enzyme activity was reduced by ~56.1% in cells that had been cotransfected with miR-375 mimic and pGL3-ERBB2, as compared with pGL3-ERBB2-Mu (P<0.01). These results indicate that miR-375 suppresses gene expression by binding to the seed sequence at the 3′-UTR of ERBB2, thus, ERBB2 may be a direct target of miR-375.

### miR-375 regulates endogenous ERBB2 expression in human gastric cancer cells

Although ERBB2 was identified as a target gene for miR-375, whether miR-375 regulated endogenous ERBB2 expression was unknown. Thus, BGC-823 cells were transfected with miR-375 mimic or inhibitor to determine whether the dysregulation of miR-375 expression affected endogenous ERBB2 expression. Compared with the corresponding control, the level of ERBB2 protein expression was significantly suppressed by miR-375 mimic and upregulated by miR-375 inhibitor (Fig. 2D).

### miR-375 overexpression suppresses gastric cancer cell proliferation

To further investigate whether miR-375 exhibits tumor-suppressive functions by targeting ERBB2, the effect of ERBB2 on miR-375-mediated cell proliferation was investigated.

BGC-823 cells that had been transfected with miR-375 demonstrated a lower capacity of proliferation compared with cells that had been transfected with the miRNA control, indicating that miR-375 suppresses gastric cancer cell proliferation. When the cells were cotransfected with the ERBB2 expression vector and miR-375 mimic, the cell proliferation ability was partially restored, as compared with the control, indicating that miR-375 overexpression mediates the suppression of cell growth through inhibiting ERBB2 expression.

## Discussion

miR-375 was first identified in murine pancreatic β-cells and the expression was also shown to be enriched in human pancreatic islet cells. However, miR-375 is not a tissue specific molecule, as it has also been detected in other tissues, including the brain and lungs, where it is important for maintaining normal function.

Low expression levels of miR-375 were first reported by three studies in 2010 (9,13,14), which hypothesized that miR-375 was associated with gastric carcinogenesis. In the present study, the expression levels of miR-375 were detected in 30-paired cases of normal and gastric cancer tissue samples. The results demonstrated that miR-375 was downregulated in almost all the gastric cancer tissues. Notably, the expression level of miR-375 was significantly lower in ERBB2-positive gastric cancer tissues as compared with ERBB2-negative gastric cancer tissues. In addition, miR-375 was shown to suppress ERBB2 expression by directly targeting the 3′-UTR of ERBB2, and overexpression of miR-375 was shown to partially inhibit gastric cancer cell proliferation through the ERBB2 pathway.

ERBB2 is a member of the EGFR family that is associated with increased proliferation of tumor cells. Between 6 and 35% of patients with gastric and gastroesophageal junction cancers exhibit ERBB2 gene amplification and protein overexpression (3). ERBB2 expression is also an important predictor of gastric cancer patient classification, which is used for further specific therapies. However, defined by immunohistochemistry, ERBB2 quantification remains imprecise. The results of the present study indicate that ERBB2 expression level detection, associated with quantified miR-375, may be used to enhance the accuracy of clinical gastric cancer classification (16).

In conclusion, the present study has partially clarified the associations between miR-375 and ERBB2-positive gastric cancer. To the best of our knowledge, this is the first study to demonstrate that miR-375 is associated with ERBB2-positive gastric cancer. Therefore, miR-375 is a candidate tumor suppressor miRNA molecule in gastric cancer and may be a potential clinical classification marker and therapeutic target for human gastric cancer.

## Figures and Tables

**Figure 3:**
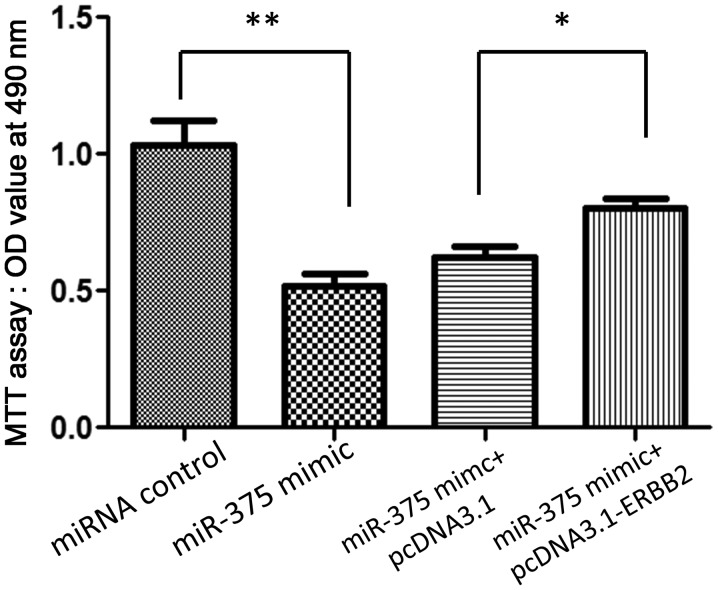
Gastric cancer cell proliferation was partially suppressed by miR-375 through targeting ERBB2. Cells were transfected with miR-375 mimic or miR-375 mimic plus pcDNA3.1-ERBB2, with miRNA control and miR-375 plus pcDNA-3.1 as controls. The MTT method was used to detect the cell proliferation ability. ERBB2, human epidermal growth factor receptor 2; miR-375, microRNA-375. ^*^P<0.05, ^**^P<0.01.
